# Experimental and Computational Methodology for the Determination of Hydrodynamic Coefficients Based on Free Decay Test: Application to Conception and Control of Underwater Robots

**DOI:** 10.3390/s19173631

**Published:** 2019-08-21

**Authors:** Juan S. Cely, Roque Saltaren, Gerardo Portilla, Oz Yakrangi, Alejandro Rodriguez-Barroso

**Affiliations:** Centro de Automática y Robótica, Universidad Politécnica de Madrid, C/José Gutiérrez Abascal 2, 28006 Madrid, Spain

**Keywords:** system identification, systems modelling, hydrodynamics coefficients, unmanned underwater vehicles

## Abstract

Hydrodynamic coefficients are essential for the development of underwater robots; in particular, for their design and navigation control. To obtain these coefficients, several techniques exist. These methods are usually experimental, but, more recently, some have been designed by a combination of experiments with computational methods based on Computational Fluid Dynamics (CFD). One method for obtaining the hydrodynamic coefficients of an ROV (Remote Operated Vehicle) is by using an experimental PMM (Planar Motion Mechanism) or CWC (Circular Water Channel); however, the use of these experimental infrastructures is costly. Therefore, it is of interest to obtain these coefficients in other ways, for example, by the use of simple experiments. The Free Decay Test is an ideal type of experiment, as it has a low cost and is simple to implement. In this paper, two different free decay tests were carried out, to which three different methods for obtaining coefficients were applied. They were compared with results obtained by CFD simulation to conduct a statistical analysis in order to determine their behaviours. It was possible to obtain values of the drag and added mass coefficients for the models analysed, where the values were obtained for an Underwater Drone Robot (UDrobot).

## 1. Introduction

The development of an underwater robot is a scalable process that starts with the conception, design, construction, control and start-up of the prototype. In every phase, the most critical and complex aspect is obtaining hydrodynamic coefficients (added mass, drag force, and linear and nonlinear term coefficients) with values as close as possible to those in real operations. This article aims to contribute to the solution of this problem based on the determination of the coefficients through the experimental method of free decay test, using scale prototype models and similitude laws. The benefits of these type of methods are that they use low-cost sensors and printed scale models, which are easily manufactured, and that they provide a good approximation of the real-size prototype, according to results that we obtain them. The behaviours of underwater rigid bodies have been studied in a recurrent manner using known geometric shapes, such as spheres, cones and prisms. When a robot has a known geometric shape, the parameters can be obtained following the Li method [1]. When the shape is not known, a better analysis is required, such as that detailed by Prestero in his Master’s thesis [2], where he recapitulated theoretical expressions for obtaining hydrodynamic coefficients (HC). However, these algebraic expressions are complex and sometimes difficult to solve; in response, Robles [3] proposed a theoretical reduction for the obtaining the hydrodynamic coefficients.

The importance of a dynamical model is in modelling the robot’s behaviour in an underwater environment and generating control systems for orientation and localisation, as shown in the work by the authors of [4]. When it is required to implement a control system independently of a physical model, it is necessary to develop more robust control systems [5]; in this article, we will not speak about this theme due the objective of this article is to improve the physical model then implement different control strategies. The different coefficients depend on whether a model allows for effects on other axes generating control problems solved by Garcia [6] using control methods based on the robot mathematical model. Gibson proposed a model for robots according to actuators on the tail of the robot, similar to the fins of fish [7]. However, obtaining these coefficients was limited to the capacity of the instrumentation of the system [8]. When the effects were crossed, it required certain identifications which allowed it to obtain the values. In the methodology of the method proposed by Avila, an oscillatory movement was used to obtain the crossed coefficients. However, this required a larger infrastructure and more complex postprocessing [9]. Another special case occurs when the model has more than one body, such as in the work by the authors of [10]. Crossed effects appeared when a robot had accessories, such as a a robotic arm, on itself; in this case, the arm dynamical crossed effects must be considered [11]. If the coefficients of rotation need to be obtained, the method proposed by Lin [12] can be applied. This is very useful when controlling rotational articulations underwater, or in an UVMS (Underwater Vehicle-Manipulator System) as defined by Antonelli [13].

The most popular way to find hydrodynamical coefficients is by conducting tests in a PMM (Planar Motion Mechanism) based on mobility in a certain direction, propelling the robot prototype through water at certain velocities and measuring the reaction force. These values are then compared with respect to the theory; see the works by the authors of [14,15]. Validation of the model can also be carried out with respect to simulations using Computational Fluid Dynamic (CFD) simulation software [16,17], which requires a correct simulation configuration [18]. Another way to use the PMM for identification is by turning on the robot’s actuators and finding the relationship between the energy input and the force and torque in the output generated by the robot [19]. Xu carried out tests in PMM and circular water channel (CWC) environments, validating the values of hydrodynamic coefficients for the crossed effect [20] and proposed new methods for the modelling of underwater nonlinear systems [21].

An alternative for obtaining the hydrodynamic coefficients is the free decay test. The free decay test is an easily implemented experiment with simple instrumentation and lower postprocessing requirements. Cruz proposed a methodology for obtaining the added mass values of bodies with known geometries and comparing them with theoretical values [22]. Nevertheless, the added mass phenomenon is a difficult theoretical achievement and requires specialised software and a complex configuration [23]. Another type of free decay test uses the dynamical behaviour of an underwater pendulum. This test has been used widely by researchers to obtain hydrodynamic coefficients of scaled models. Ross used this method, in 2004, to obtain hydrodynamic coefficients; Professor Fossen was part of this team [24]. The use of scaled models allows obtaining the coefficients using smaller pools and scale these results up for bigger models [25]. Chin validated this method using CFD and numerical tools to obtain convergence between the data [26]. These models allow obtaining values for the added mass and drag coefficients, which was the origin of their popularity [27].

In this paper, we compare the free decay experimental and free decay pendulum tests in which the hydrodynamic coefficients of a robot with nonregular shape are obtained. The identification process uses three different methods to obtain the different coefficients necessary for modelling hydrodynamical behaviour. The obtained results are compared to determine the final value of coefficients for the robot hydrodynamic model. The different kinds of free decay tests can be used for scaled models when the required infrastructure to obtain the full scale values is not available, and can serve as an alternative for research groups to experimentally determine the values of the hydrodynamic coefficients of underwater bodies. The main characteristics of this method are the implementation and lower instrumentation costs in the process of development of an underwater robot. The paper is organised as follows. The dynamical model for an underwater robot is given in Section 2, where we briefly describe the robot, the present hydrodynamic effects in the cases under investigation and how to quantify them. In Section 3, the methodology for obtaining values, the experimental description and a brief summary of the obtained results are presented. In Section 4, the stochastic results, first with respect to the CFD simulation and then with respect to the test results, and the comparison between them, are shown. A discussion of the results is given in Section 5. We finish with conclusions and references.

## 2. Dynamic Model of a Underwater Drone Robot

Underwater robot modelling is different to modelling a robot on the earth’s surface, due to the forces generated by the fluid (in this case, water). Everything submerged and moving in a fluid is subjected to specific hydrodynamic effects, such as added mass or drag, which are described in this section. Another requirement for modelling a robot in an underwater environment is a full description of its geometric, material and physical characteristics, which will be described in this section, as well.

### 2.1. Underwater Robot Modelling

The dynamical model can be obtained from the work by the authors of [28], here given as Equation (1), where υ is the vector of body velocities, η is the attitude of the robot, M${}_{RB}$ is a rigid body matrix, C${}_{RB}$ is the Coriolis matrix, g(η) is the gravitational component as a function of the robot’s attitude with respect to the world frame, τ is the input force vector and F${}_{h}$ is the component of hydrodynamic forces, from which one obtains the hydrodynamic coefficients, as shown in this article.

(1) $$M_{RB}\dot{\upsilon }+C_{RB}\left(\upsilon \right)\upsilon +g\left(\eta \right)=\tau -F_{h}.$$

**Figure 1:**
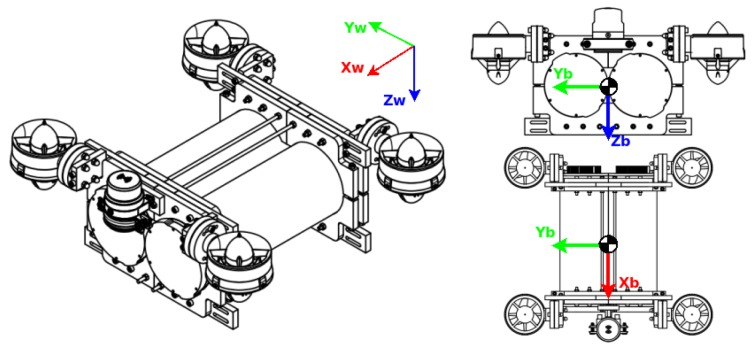
Visualisation of the centre of mass (com) from two different points of view. Subindex ”w” means with respect to the world frame, while subindex ”b” means with respect to the body frame.

The velocity vector (υ) is with respect to the body frame, while the attitude of the robot (η) is with respect to the world frame. The hydrodynamic effects are all referred to with respect to the body frame, except for the buoyancy, which is referred with respect to the volumetric centre or barycentre. In Figure 1, the location of the mobile reference frame (in the centre of mass of the robot) and how it moves with the robot are shown.

### 2.2. Effects Hydrodynamics

The hydrodynamics have an effect only on the movement of a robot; however, the buoyancy has an effect on an underwater body at all times. According to Fossen [28], the Coriolis matrix does not have values when the movement is rectilinear. The expression describing the hydrodynamic effects is shown in Equation (2), where $M_{A}$ is the added mass, $C_{A}$ is the added Coriolis, *D* is the drag coefficients values and *g* is the gravitational effect.

(2) $$F_{h}=M_{A}\dot{\upsilon }+C_{A}\left(\upsilon \right)\upsilon +D\left(\upsilon \right)\upsilon +g\left(\eta \right).$$

#### 2.2.1. Added Mass

The definition of added mass, according to the authors of [29], is the equivalent effect of a phenomenon if the model has additional mass in its dynamical model. The value of the generated force by the added mass is proportional to the acceleration multiplied by a factor that depends on the body’s volume and its geometry. As shown in Equation (3), this is a case where there are three degrees of freedom. In Equation (3), *Cm*${}_{i}$ is the added mass coefficient for each axis, ρ is the fluid density and *V* is the submerged volume. This representation is ideal because, in the real case, there are cross effects.

(3) $$M_{A}=\left[\begin{array}{ccc} \rho Cm_{x}V & 0 & 0\\ 0 & \rho Cm_{y}V & 0\\ 0 & 0 & \rho Cm_{z}V \end{array}\right].$$

According to Fossen [28], if a robot is symmetric on its planes and moving with low velocity, the added mass coefficient can be considered equal to the mass of the robot; that is, the value of added mass coefficient times robot mass equals 1. Nevertheless, for robots with nonsymmetric geometries, this does not happen and those values must be obtained, in either a theoretical or experimental manner.

#### 2.2.2. Damping Forces

The damping forces are a component of the hydrodynamic forces that emulate a force in the reverse sense of the movement, as a function of the velocity. The literature has proposed different sources of this phenomenon (see the works by the authors of [28,30]); in this paper, we concentrate on damping due to vortex shedding. Damping due to vortex shedding is based on the D’Alembert paradox, from which it was deduced that frictional forces are present in a viscous fluid, and so the system is not a conservative system with respect to the energy. Its modelling is known, due to being a component of the Morison equation, where ρ is the fluid density, $C_{d}$ is the drag coefficient, *A* is the area projected perpendicularly to the body velocity and *u* is the body velocity, as shown in (4).

(4) $$F_{D}=\frac{1}{2}\rho C_{d}A\left|u\right|u.$$

It has been considered that all effects can be modelled with linear and quadratic coefficients. However, it is complicated to separate these effects; in the work by the authors of [31], hydrodynamic damping is commonly referred to as a combination of linear and nonlinear effects, which can be seen in (5).

(5) $$D\left(\upsilon_{r}\right)=D+D_{n}\left(\upsilon_{r}\right).$$

#### 2.2.3. Buoyancy

The buoyancy effect, which is defined by the Archimedes principle, is not part of the hydrodynamic coefficients, but is a hydrostatic effect that is applied to the centre of the volume. If the mass of a robot is distributed in a homogeneous way, the centre of volume coincides with the centre of mass. The effect on the model depends on the attitude of the robot.

## 3. Methodology and Experimental Frame

**Figure 2:**
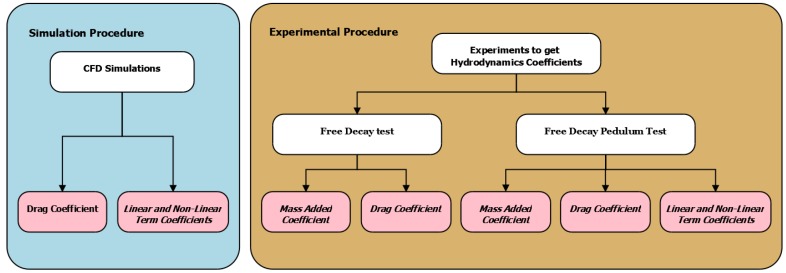
The proposed methodology for obtaining the hydrodynamic coefficients from the experimental procedure. On the right side, the two experiments based on free decay tests are shown. On the left side, a description of the simulation procedure to obtain the values for the drag coefficient and linear and nonlinear term coefficients is shown.

The methodology selected to obtain the hydrodynamic coefficients is based on the experimental procedure of free decay tests. These tests can obtain the coefficients from a scaled model or from a real-size model. The theoretical values for validation by comparison were obtained by CFD simulation. Figure 2 shows the work flow for obtaining different coefficients. For each experiment (simulated or test), we will obtain values for the drag coefficient, but only the real experiments will obtain values for the mass added coefficient. In Figure 2, the light blue square describes the simulation procedure, while the dark orange square describes the experimental procedure.

The proposed procedure starts with a CFD simulation, which obtains the coefficients for the drag force. These coefficients may be obtained in two ways: The first way is using the Morison equation to obtain $C_{D}$, and the other way is using the Chin method [26] to the obtain linear ($K_{L}$) and nonlinear term coefficients ($K_{Q}$). CFD simulation is an option in the research process for finding an indicator or an estimated value for a target value, not a real or definitive value. The CFD result values are useful in comparison to the experimental values obtained.

In the experimental procedure, the free decay test was carried out under two modalities: one was a free decay test with a recovery force, such as a spring force; the other one was a free decay test where the recovery forces were provided by pendulum dynamics. Both tests were done in underwater conditions. The coefficients obtained experimentally were the mass added coefficient and the drag coefficient ($C_{D}$), both of which were obtained in each of the two tests. The linear and nonlinear term coefficients were obtained only in the free decay pendulum test.

### 3.1. Prototype of the Underwater Robot

**Figure 3:**
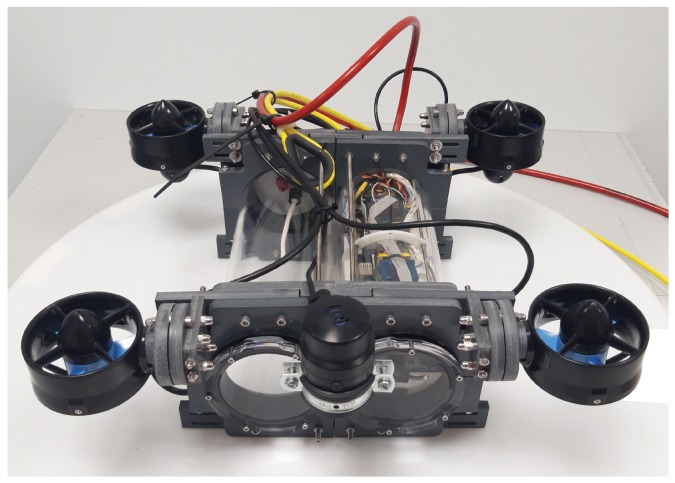
The underwater drone robot. The right enclosure is used for the robot’s movement and navigation electronics, and the left enclosure is used for the waterproof payload (e.g., the electronics for a robotic arm).

The modular underwater robot was an ROV with the shape of a drone, designed and built by the Research Group of the Robots and Intelligent Machines in the Centre for Automation and Robotics, it was named Underwater Drone robot (UDrobot). The robot has three main components: chassis, cylinder enclosures, and thrusters. The chassis was composed of two frames. Connected between these, in the middle, there were the two cylinder enclosures. Two thrusters were located on each frame of the chassis. The robot can be seen in Figure 3.

The robot has two cylinder enclosures, where the left enclosure contains the electronics for the robot’s control and the right enclosure carries electronics or other accessories. The thruster was a T-200 reference of the Bluerobotics brand, with a nominal voltage of 12 volts and a thrust of 34.3 N.

**Figure 4:**
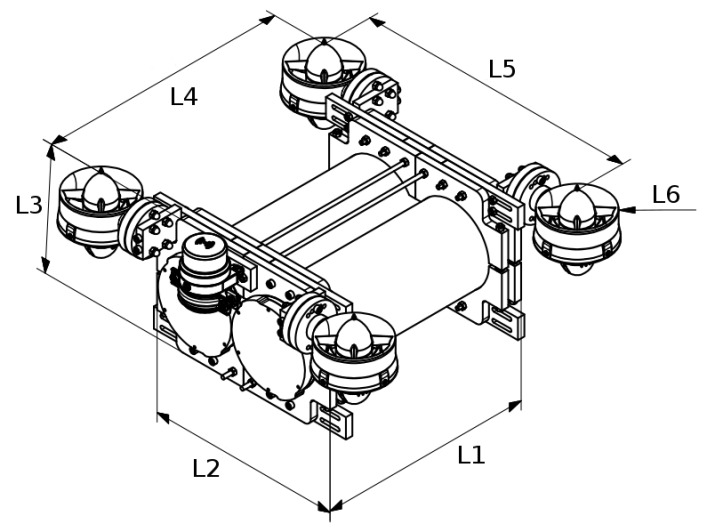
In this schema, the lengths of the robots can be seen. These measurements take into account the dimensions of the experimental models. The values for the real robot are shown in Table 1.

With respect to the sensors, the robot had a sonar Micron from the Tritech company located on its front upper part. This was the only sensor located outside the enclosures. Inside of the enclosures, the electronic parts and sensors for the operation of the robot were located. The convention for the reference system is shown in Figure 1. The assignment for the reference system is in accordance with the SNAME notation. The dimensions of the robot were required for the generation of its physical model, the most relevant characteristics being the mass and volume. The robot length and size are related in Figure 4 and Table 1.

### 3.2. Test-Bed for the Estimation of Hydrodynamic Parameters Based on Free Decay

**Figure 5:**
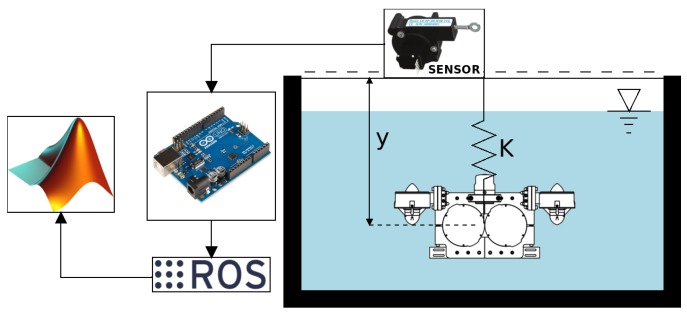
The scheme used for the free decay test, where *y* is the length displacement of the model when it is oscillating and *K* is the spring constant. The sensor is a linear encoder, from which the information is sent to the Arduino, which records the data using the ROS framework. Afterward, the information is processed using the Matlab software.

The free decay test is a test carried out on a rigid body which oscillates thanks to a spring [22]. The rigid body, in this case, was the scaled robot model. The schema is described in Figure 5, where the different elements which are part of the test are shown.

**Figure 6:**
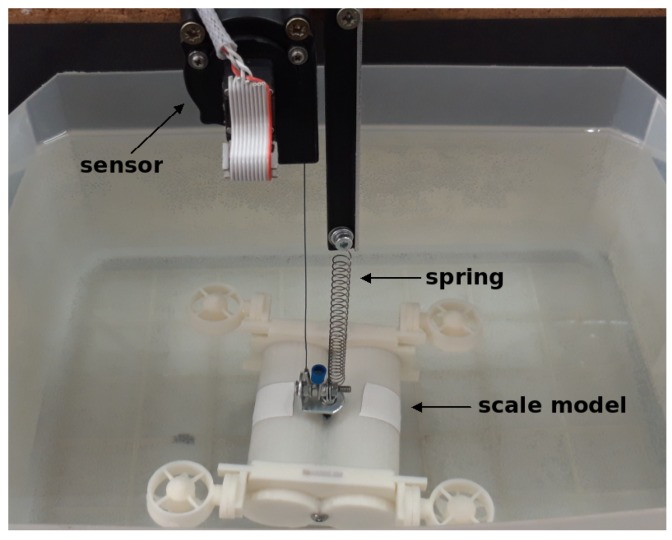
The values for the free decay test are obtained using the recovery force from a spring. The parameters required for obtaining the values used in this case are shown in Table 2.

The final implementation of the schema is shown in Figure 6, where the sensor on the top can be seen, the spring holds the model and the scaled model is placed in the water for testing. The experimental procedure to obtain the values using the free decay test is based on the diagram in Figure 5.

This experiment models the dynamical behaviour in underwater conditions, considering inertial mass effects and nonlinear drag. The motion equation is described in (6), which was obtained from the Morison equation [32].

(6) $$\left(m+m^{\prime }\right)\ddot{y}+c\dot{y}+\frac{1}{2}\rho C_{D}A\left|\dot{y}\right|\dot{y}+ky=0.$$

This experiment allows for direct extraction of the values of added mass and a drag coefficient from the motion, as was shown in the work by the authors of [32].

#### 3.2.1. Test Set-Up

The experiment was based on the dynamical behaviour of an underwater mass–spring system. The model used is shown in Figure 6, and it had the physical characteristics shown in Table 2. The model material was acrylonitrile butadiene styrene (ABS) plastic, which contains lead to increase its mass.

The spring that holds the model to the fixed surface had a constant of K=63.6$\frac{N}{m}$, as was used for the same spring in the work by the authors of [33]. The container in which the test was made measured 30 cm wide by 40 cm deep. The sensor that was used for measuring the distance was an LX-EP-40 with a resolution of 2.45 count/mm and accuracy of 0.4 mm/count. The process of the experiment consisted of six launches for each one of the three axes with four different initial positions (10 mm, 20 mm, 30 mm and 40 mm), which produced a total of 24 launches for each axis. The initial position was taken as reference value for determining the measurement of the stabilised position. The information obtained from the sensor was processed on an Arduino Board and was sent on a serial port at 115,200 baud. The configuration and serial port administration were made by ROS, later to be processed in Matlab. The identification parameters were obtained for the identification method, which will be discussed in the next section. With the obtained values, a statistical process was performed to determine their validity in the identification process.

#### 3.2.2. Identification Method

The solution for the dynamical model of the mass–spring system is shown in Equation (6). It has solution in the time domain as a function of two coefficients that determine the dynamical behaviour. The first coefficient is the natural frequency ($\omega_{n}$), the value of which is defined in (7), where *K* is the spring constant, *m* is the solid mass and $m^{\prime }$ is the added mass. If the natural frequency of the system is known, we can obtain a value for the added mass.

(7) $$\omega_{n}=\sqrt{\frac{K}{m+m^{\prime }}}.$$

**Figure 7:**
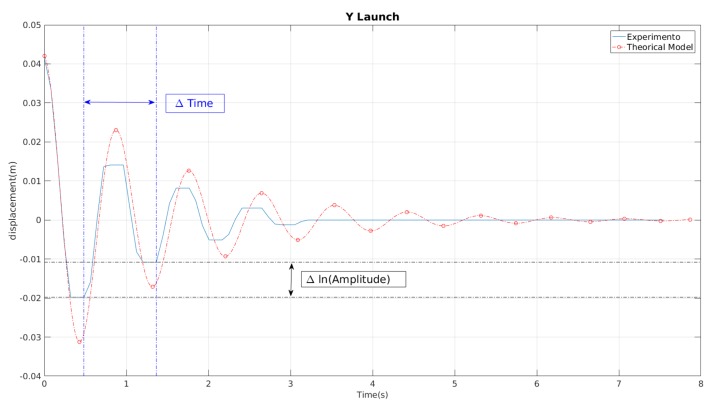
The behaviour of a launch on the *Y*-axis for 4 cm of amplitude. The value of Δ (time) is the distance between two consecutive peaks. The value of Δ (ln(Amplitude)) is the logarithmic variation between the two values. The blue line is the result of the experiment and the red line is the response of the reconstructed model from those values.

In the graphical form, the natural frequency is obtained by measuring two continuous peaks of the position response with respect to time. As this value has to be converted to radians, it is multiplied by 2π. It can seen in Figure 7. The other coefficient that determines the response of the mass–spring system is the damping coefficient (ς), according to [34]. For this case, the system has a composed ς. In accordance with [32], the reason for this is that the mechanical system has a coefficient and the water effect adds another damping coefficient, as defined in Equation (8), where $\varsigma_{s}$ is the mechanical system damping coefficient and $\varsigma_{f}$ is the damping coefficient due to the fluid.

(8) $$\varsigma =\varsigma_{s}+\varsigma_{f}.$$

Obtaining $\varsigma_{f}$ numerically does not contribute to the identification process of the hydrodynamic coefficient. However, the value of $\varsigma_{f}$ can be written in terms of a drag coefficient ($C_{D}$) of a model with just the applied damping due to vortex shedding [32]. In Equation (9), it is related the values of $C_{D}$ and $\varsigma_{f}$, where ρ is the fluid density, *D* is a dimensional measurement of the submerged model, *m* is the mass, $m^{\prime }$ is the added mass and A the initial launch amplitude.

(9) $$\varsigma_{f}=\frac{\rho D^{2}}{4\pi (m+m^{\prime })}\frac{8}{3}C_{D}\frac{A}{D}.$$

The value of the obtained ς can be deduced from the logarithmic decrease amplitudes of the rigid body position in the fluid, as can be seen in Figure 7; this method was used in the work by the authors of [34]. The value of $C_{D}$ can be obtained from the value of the damping coefficient in the fluid $\varsigma_{f}$, as can be seen in (8).

**Figure 8:**
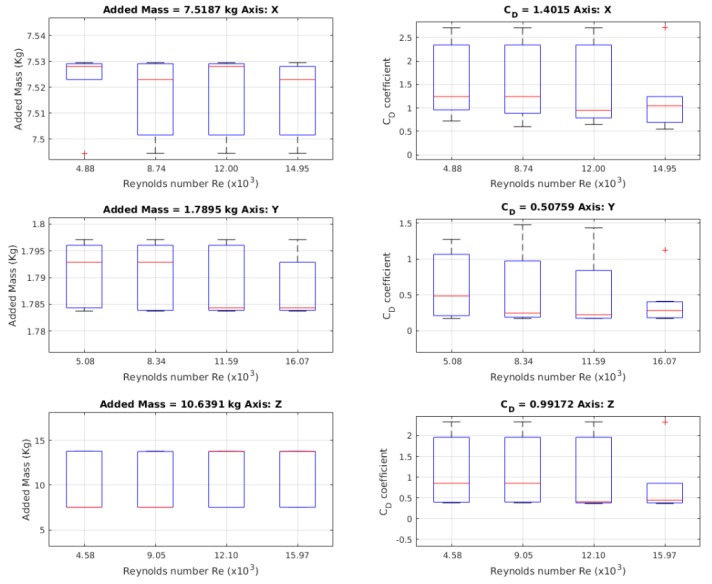
Values for the added mass obtained using a free decay experiment by least squares as in the Chin method. On the left, the results for the values for added mass are shown. On the right, the values of drag coefficients are shown.

In Figure 8, how we obtained the drag coefficients with respect to the velocity is shown. The value of $C_{D}$ for the *X*-axis is 1.4015. For the *Y*-axis, the value of $C_{D}$ is 0.50759. The value of $C_{D}$ for the *Z*-axis is 0.99172. The values marked in red were outliers; these values were not taken into account. The values for the velocity are represented in Reynolds Number for comparison with respect to a differently sized model.

### 3.3. Test-Bed for the Estimation of Hydrodynamic Parameters Based on Pendulum Experiments

The free decay pendulum test is an experiment that allows finding the hydrodynamic coefficients of a body oscillating in pendulum-like way in a fluid [26]. In the experiment, the angular acceleration, angular velocity and model angular position must be known.

#### 3.3.1. Test Set-Up

**Figure 9:**
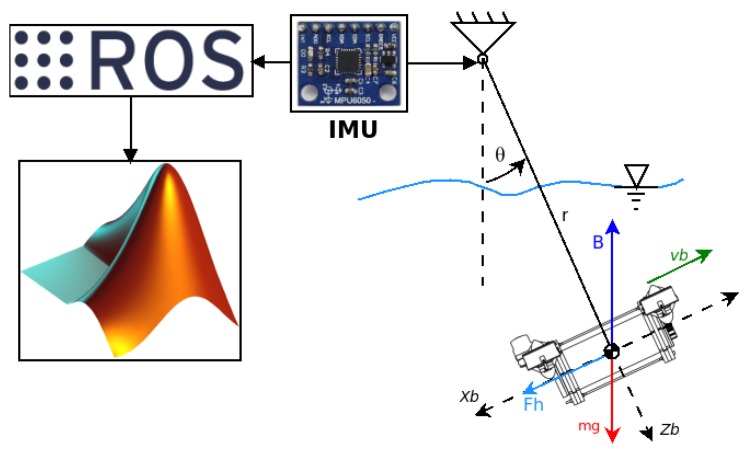
On the right, the free body diagram used for modelling the body oscillating underwater is shown, where Θ is the angle; r is the pendulum length; B is the buoyancy; *m* is the mass; g is the gravity acceleration (9.79 $\frac{m}{s^{2}}$); v${}_{b}$ is the body velocity; X${}_{b}$ and Z${}_{b}$ are the axes of the reference body system, respectively; and F${}_{h}$ is the hydrodynamical force. The angle is measured by an IMU, which sends the info to ROS, after which it is processed in Matlab.

**Figure 10:**
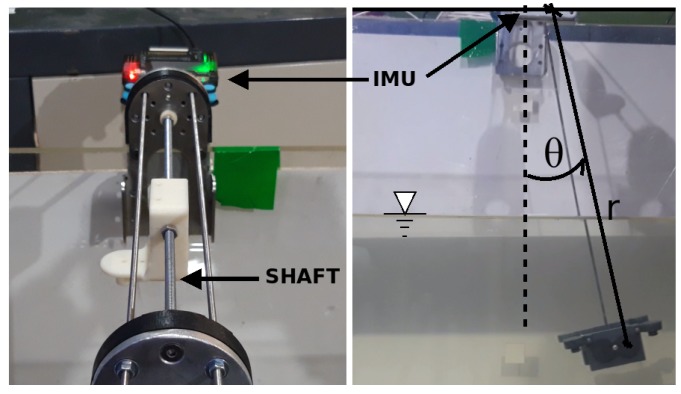
Diagram of the free decay pendulum test, where θ is the angle with respect to the stability point and L is the length of the pendulum.

The general experiment schema is shown in Figure 9, where *r* is the pendulum length and Θ is the model angle with respect to the equilibrium condition. The added mass, linear and quadratic damping coefficients can be obtained in this experiment. This kind of model was proposed in works by the authors of [28,31]. The model used in this experiment is shown in Figure 10, where it is possible to see the implementation of the IMU and the rotation shaft.

The experiment is based ion the pendulum performance. The pendulum mass is a scale model, which was different for this experiment; its characteristics are shown in Table 3, where the model material was PVC.

The sensor used for obtaining the angle was an IMU with reference MPU6000. The angle estimation was made by a Kalman filter inside of the Ardupilot firmware, with a sample time of 10 milliseconds. The information was sent using the MAVLink protocol. The information was stored and managed by ROS, and afterwards it was processed in Matlab. The experiment protocol was to do six launches, each separated by 30 s. That process was repeated for each axis, with for initial different angles (2.5, 5, 10 and 15 degrees), resulting in 24 launches per axis in total. The angle measurement used, as reference value, the stabilisation position of the pendulum, in a levogiro sense. The hydrodynamic parameters obtained are shown in the next section. The obtained values were processed stochastically to determine the most relevant values.

#### 3.3.2. Identification Method

The dynamical model was based on the free body diagram shown in Figure 9, where the hydrodynamical forces were modelled as a function on linear and quadratic coefficients, as in (5). An identification method was used [35] for the model obtained by the linear and nonlinear term coefficients. A method using the Morison equation was implemented to obtain the drag coefficient and added mass, in order to compare the results of different models.

The dynamical model of an oscillating body is based on the force summation with respect to the centre of mass of the robot, as shown in Equation (10).

(10) $$m\ddot{x}=B\sin\left(\Theta \right)-mg\sin\left(\Theta \right)-F_{h}.$$

The hydrodynamical force ($F_{h}$) is defined in (11). The difference with respect to the work by the authors of [35] is the use of the Morison equation, meaning expressing the force as the function of quadratic coefficients without linear term coefficients. This is proposed to consider the changes most relevant the quadratic value, in order to compare the results between the two kinds of free decay tests.

(11) $$F_{h}=m^{\prime }\ddot{x}+K_{D}\left|\dot{x}\right|\dot{x}.$$

Combining Equations (10) and (11), where $\dot{\Theta }=r\dot{x}$ and *r* is the length pendulum, we get (12):

(12) $$\left(m+m^{\prime }\right)r\ddot{\Theta }=\left(B-mg\right)\sin\left(\Theta \right)-K_{D}r^{2}\left|\dot{\Theta }\right|\dot{\Theta }.$$

Considering $m^{\prime }=\rho C_{m}V$, where ρ is the fluid density, $C_{m}$ is a mass coefficient and *V* is the body volume, reorganising the equation gives

(13) $$\ddot{\Theta }=\frac{B-mg}{m+\rho C_{m}V}\sin\left(\Theta \right)-\frac{K_{D}r}{m+\rho C_{m}V}\left|\dot{\Theta }\right|\dot{\Theta }.$$

The difference, with respect to the method of the authors of [35], is the amount of hydrodynamical coefficients considered for each coefficient obtained, where a${}_{1}$ = $\frac{B-mg}{m+\rho C_{m}V}$ and a${}_{2}$ = $\frac{K_{D}r}{m+\rho C_{m}V}$. This will give us, as a result, the reduced expression

(14) $$\ddot{\Theta }=a_{1}\sin\left(\Theta \right)-a_{2}\left|\dot{\Theta }\right|\dot{\Theta }.$$

As scale models were used, the solution of Equation (14) requires us to apply similarity laws. The values for added mass ($m^{\prime }$), in Kg, are shown in Equation (15). The value of $C_{D}$, where $A_{wet}$ is the area of the crossed section, is shown in Equation (16).

(15) $$m^{\prime }=\frac{\rho Vg-mg}{a_{1}}-m.$$

(16) $$C_{D}=\frac{2a_{2}}{\rho A_{wet}}.$$

**Figure 11:**
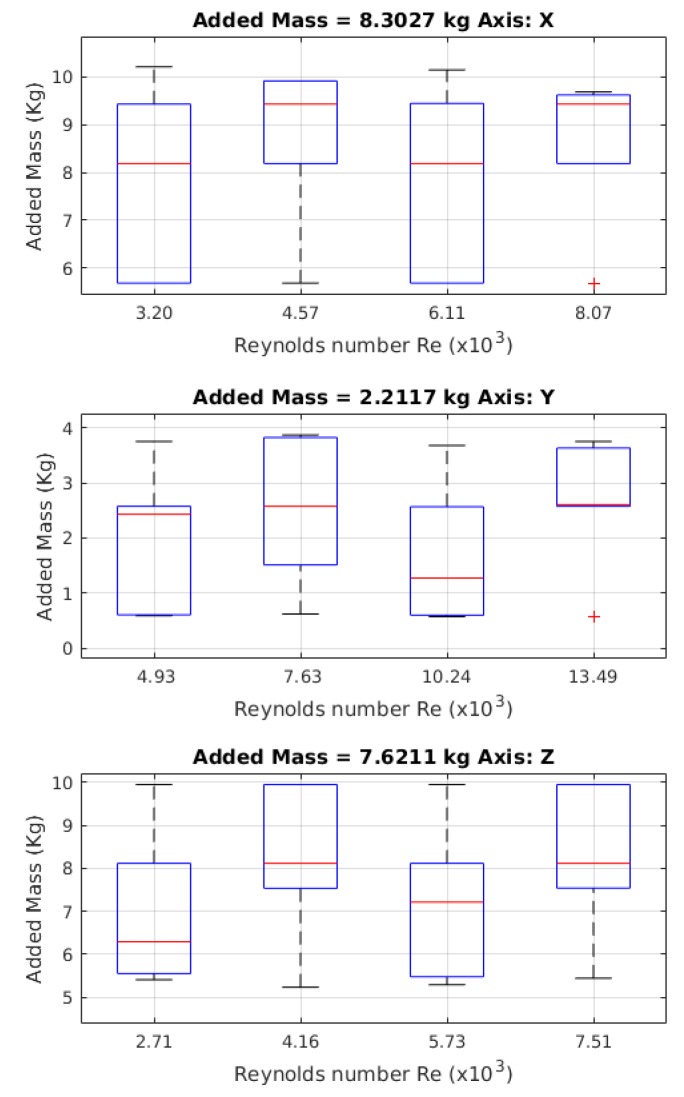
Values obtained for the mass added using the free decay pendulum test. The values of the red crosses are outliers.

In Figure 11, the results for the added mass values are shown, where 24 launches were made, one for each different initial position, allowing the model to reach different maximum velocities that gave different Reynolds numbers.

**Figure 12:**
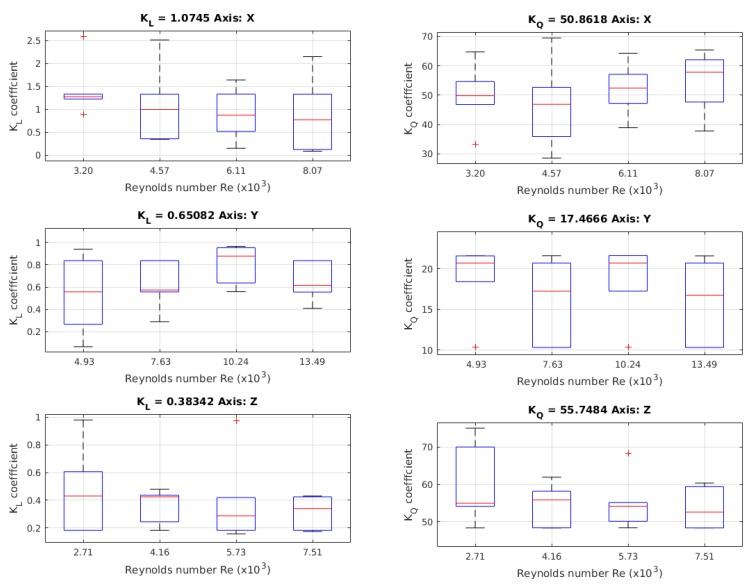
Values of the linear coefficient obtained in the free decay pendulum test. This value is not present in the Morison equation, but is added to take into account other hydrodynamic phenomena.

In Figure 12, the results for the linear coefficient *K*${}_{L}$ are shown, where, for every axis, 24 launches were made. Additionally, it shows the results for the coefficient *K*${}_{Q}$, where, for every axis, 24 launches were made with different initial positions, generating different maximal velocities. The values were obtained using the Chin method.

**Figure 13:**
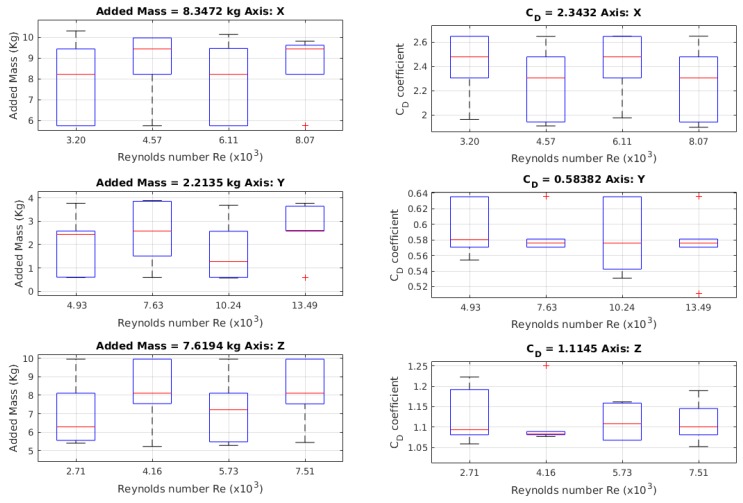
Values for the added mass using the Morison equation for the free decay pendulum test. This is a novel implementation to obtain these values for this kind of test.

In Figure 13, the added mass results obtained through the method described in this paper are shown, and Figure 13 shows the results for drag coefficient $C_{D}$ found using the described method in this article. The values for the drag coefficient were obtained from another coefficient, $K_{D}$, which is the full value of the drag part of the Morison equation.

### 3.4. Estimation of Hydrodynamic Parameters Based on Simulation

A common way to find the hydrodynamic coefficients for a body submerged in the some fluid is by using Computational Fluid Dynamics (CFD) software, because it shows the hydrodynamic behaviour of the bodies. CFD simulation needs to be configured by defining speeds, pressures, temperatures, displacement sense and fluid properties.

In this work, the CFD results were useful for comparing the testbed results, in order to validate their results. The hydrodynamic coefficients of the robot cannot be obtained theoretically due to the irregularity of the robot’s shape. The coefficients obtained through the simulation were found using the Autodesk CFD software. The analysis was made with velocities ranging between 0.2 and 10 $\frac{m}{s}$; translated into Reynolds numbers between 0.6 and 30.3 [28].

#### 3.4.1. CFD Set-Up Parameters

The environment analysed was in the computational domain and its configuration depended on the model analysed and on boundary conditions. The CAD model was simplified, while preserving the more representative measurements and geometries, and it was placed at the centre of the simulation volume. The domain needed to be large enough to ensure that the distance between the domain walls and the model did not affect the hydrodynamic behaviour.

Mesh configuration: The grid size is significant in the process of obtaining the hydrodynamic coefficients because the relationship between accuracy and efficiency is inversely proportional to the grid size, but directly proportional to the computational cost of the simulation. We selected 3 referents measurements to set the mesh size, where the measurement of reference for the X axis was L5, for the Y axis was L4 and for the Z axis was L3; all measurements can be visualised on the Table 1. The chosen mesh form was a rectangular prism, which was 5.94 *m* wide or 15 times L5, 2.31 *m* high or 13.66 times L3, and 5.5 *m* long or 15 times L4.

**Figure 14:**
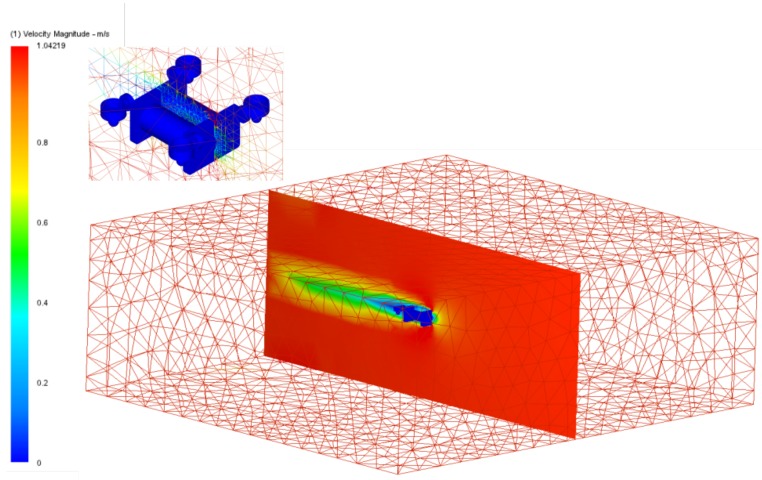
The mesh used in the simulation of the Autodesk CFD software. The size corresponds to the volume used for the fluid, which is considered to have a distance of at least 10 times the length of the robot on that axis. At the top, the variation of the triangle size in the mesh near to the robot is shown. The robot is in blue.

There were two main zones in the grid: The first is the biggest zone, in which the size of the grid was bigger due the distance between the point of the domain and the robot, as shown in Figure 14, where the red zones had values around the corresponding simulation value of 1$\frac{m}{s}$. The other zone is near the robot, and most of the calculation was concentrated on these points. Figure 14 shows changes to the fluid speed around the robot, while, in the other zone, the values were constant. The resolution factor is a factor related to the fineness of the mesh and it is according to the curvature of the model, the value used for the simulation was 1.0 being acceptable within the range of 0.1 to 3.0, where the values closest to zero generate near of curvatures a finer mesh.

In summary, the mesh configuration had a grid quality of 0.45 (grid quality indicator); if the grid quality was higher, then there was too high of a computational cost. The total number of elements was 204,258 and the total number of nodes was 46,879. The minimal refinement length was 0.00473 times L3.

Boundary conditions: The physical conditions of the fluid must also be considered; the temperature was 20 centigrade and the hydrostatic pressure was 101 MPa. According to the work by the authors of [36], *D* is a reference distance in the robot, where the distance between the walls and model must be almost 10 times *D*; this relationship is named the gap ratio. The gap ratio is defined as $\frac{e}{D}$ in the work by the authors of [32], where *e* is the distance between the model and wall, and *D* a significant robot dimension perpendicular to the wall, which is visualised in Figure 14. For this case, the most representative conditions were two walls as inlets: the first one as the velocity inlet and the opposite wall as the velocity outlet. The inlet wall could be placed 2.5 times *D* or higher respect to the model. For this case, the value of *D* was the length of the robot, L5 (Table 1), with a value of 0.395 m.

#### 3.4.2. CFD Solver Settings

The solution mode used was the transient mode, which requires time step size, time stop and the number of inner iterations. This simulation required at least 300 iterations, but was carried out with 500 iterations. The desired simulation time for each of the speeds was 50 s, at which time the 500 iterations are carried out, thus generating a time step size of 0.1 s. The calculation could be terminated when the average residuals were closest to zero.

Turbulence model used in CFD simulation: The turbulence model used in the simulation is the Wilcox’s K-epsilon (K-ϵ) model [37]. It is used in different applications and allows obtaining results with lower computational cost than the constant eddy viscosity model. The k-epsilon model is a RANS-based turbulence model that uses two transport equations. In the case of the advection scheme, the “Monotone streamline upwind” scheme has been used, which is numerically stable and is widely used when the surfaces are smooth, it is not the most precise scheme compared to other schemes such as the Petrov–Galerkin scheme and its variants.

**Figure 15:**
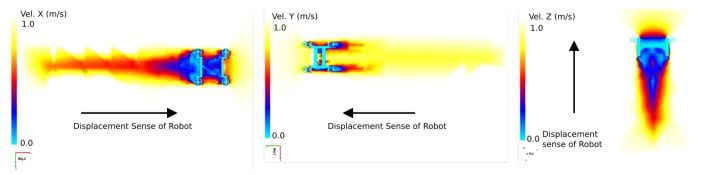
Three different results for the CFD simulation. On the left, velocity is shown in the *X*-axis. In the middle it is shown in the *Y*-axis and on the right in the *Z*-axis. These results were obtained at 1 $\frac{m}{s}$. The dark blue zones are values close to 0 $\frac{m}{s}$.

The results obtained from the CFD simulation are shown in Figure 15. These images are meaningful as they demonstrate the physical behaviour in each axis of the robot; the theory describes robot movement in terms of each axis when the robot has a complex geometry and analysis of its movement is difficult. In Table 4, a summary of the parameters used by the CFD simulation can be visualised.

## 4. Experimental Results Description

In agreement with the procedure described in the methodology, in this section, we will show the obtained results from the CFD, free decay test and free decay pendulum test, along with their comparison and a brief summary at the end. First, the results of the CFD simulation will be shown, describing the results and configuration of the environment, after which the results of each of the experiments, using specific procedures to obtain the coefficient values also described in methodology, will be shown. For a better understanding, the values found by each test were compared with the obtained coefficients using CFD. The error percent is with respect to the CFD values.

### 4.1. CFD Simulation Results

According to the simulation requirements, the mesh was configured for the simulation in CFD Autodesk with a width dimension of 5.94 m, length of 5.5 *m* and height of 2.31 m. The number of triangles increased near to the robot, such that the configuration in the mesh allowed for better values for the volume near the robot, as can be seen in Figure 14.

**Figure 16:**
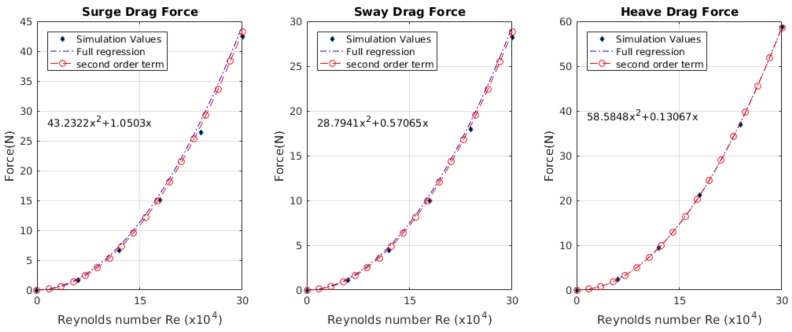
The values of the regressions obtained for the X, Y and Z axes, in terms of their surge, sway, and heave speeds, respectively. The black diamonds are the values obtained using the CFD simulation, the regression with all the terms is the dotted blue line and the red line shows the model using just the quadratic coefficient.

Figure 16 shows the obtained values of the simulation using CFD simulation. The coefficients for the drag force using the Chin method [26], where there were linear K${}_{L}$ and nonlinear K${}_{Q}$ coefficients; for the *X*-axis with a quadratic coefficient of K${}_{Q}$, a value of 43.2322 was found, and for the linear coefficient K${}_{L}$ with an R${}^{2}$ value of 0.9960, a value of 1.0503 was found. For the drag force on the *Y*-axis, the quadratic coefficient *K*${}_{Q}$ was 28.7941 and the value of *K*${}_{L}$ was 0.5706, with an R${}^{2}$ value of 0.9963. Finally, the results obtained for the *Z*-axis in the quadratic coefficient *K*${}_{Q}$ was 58.5848 and for the linear coefficient *K*${}_{L}$ was 0.1306, with an R${}^{2}$ value of 0.9967. The obtained values are summarised in Table 5.

Using the method shown in this paper, drag coefficients were obtained for the *X*-axis, a coefficient of 1.7021 with an R${}^{2}$ value of 0.9961; for the *Y*-axis, a coefficient of 0.6226 with an R${}^{2}$ value of 0.9964; and the *Z*-axis, a coefficient of 1.2929 with an R${}^{2}$ value of 0.9967. The value of *K*${}_{D}$ was the sum of the drag part in the Morison equation.

### 4.2. Free Decay Experiment Results

#### 4.2.1. Added Mass

**Figure 17:**
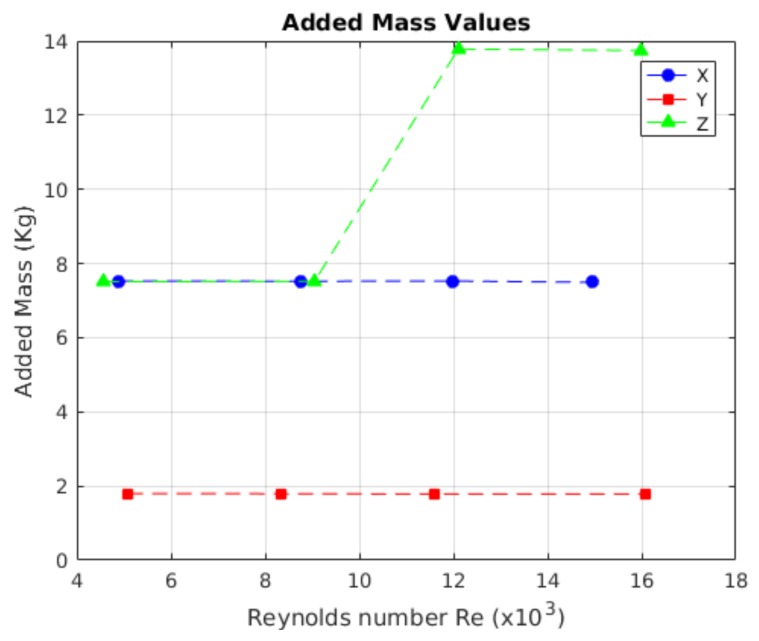
Behaviour of the added mass. The values for each axis are similar at all speeds.

From the free decay test, the added mass values and drag coefficients $C_{D}$ were obtained. In Figure 17, the results of obtaining the added mass value are shown, where, for each axis, 24 launches was made, each with a different initial position, and allowing the model to reach different maximum velocities per launch. The added mass values were related with the Reynolds number, such that they could be compared between the tests, independently of the lengths of the model. For quantifying and comparing the obtained values for the added mass, a comparative analysis between the launches was made. The comparative process was carried out one by one and the *p* values were found, by which the existence of relationships between launches could be determined. The results of the comparison are shown in Table 6.

In the work by the authors of Figure 17, the behaviour of the added mass values that were obtained for each axis are shown. The behaviour in the X- and Y-axes was preserved in almost all Reynolds numbers, while the added mass value for the *Z*-axis tended to have two values.

For the *X*-axis, the value of added mass was 7.5187 kg, with a *Cm*${}_{x}$ of 0.8172 times the robot’s mass. On the *Y*-axis, the added mass was 1.7895 kg, with a *Cm*${}_{y}$ of 0.1945 times the robot’s mass. For the *Z*-axis, the value of added mass was 10.6391 kg, with a *Cm*${}_{z}$ of 1.1564 times the mass of the robot.

#### 4.2.2. $C_{D}$ Coefficient

**Figure 18:**
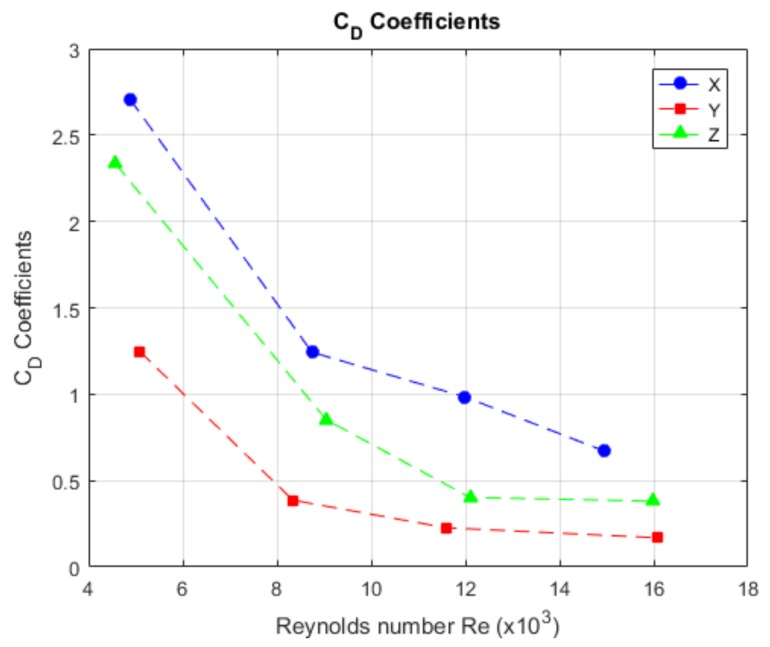
The behaviour of the value of the drag coefficient with respect to the speed in Reynolds number.

Figure 18 visualises the behaviour of the drag coefficient, according to the Reynolds number. The coefficient decreased as the velocity rose [32], which was more obvious in the *X*-axis; while, in the *Y*- and *Z*-axis, the behaviour was not too relevant. In Table 7, the obtained values for the free decay test are shown in a comparative way; furthermore, the other physical values obtained in the test, the damping coefficient and the natural frequency, are shown.

### 4.3. Free Decay Pendulum Experiment Results Using Chin Method

In the free decay pendulum test using the Chin method [26], the values for the added mass, linear coefficient *K*${}_{L}$ and quadratic coefficient *K*${}_{Q}$ were obtained, and, using the method detailed in this article, the drag coefficient $C_{D}$ and added mass were also obtained.

#### 4.3.1. Added Mass

The value for the added mass for the *X*-axis was 8.3027 kg, with a *Cm*${}_{x}$ of 0.9024 times the robot’s mass. For the *Y*-axis, the added mass value was 2.2117 kg, with a *Cm*${}_{y}$ of 0.2404 times the mass of the robot. For the *Z*-axis, the added mass was 7.6211 kg, with a *Cm*${}_{z}$ of 0.8283 times the robot’s mass.

#### 4.3.2. *K*${}_{L}$ Coefficient

The *K*${}_{L}$ value for the *X*-axis was 1.0745. For the *Y*-axis, the value of *K*${}_{L}$ was 0.6508. For the *Z*-axis, the value of *K*${}_{L}$ was 0.3834. Comparing the CFD simulation values for *K*${}_{L}$, we determined an error for the *X*-axis of 2.3%; for the *Y*-axis, the error value was 14.05%; and the error value in the *Z*-axis was 193.43%. In Table 8, the comparison between the results for *K*${}_{L}$ are shown.

#### 4.3.3. *K*${}_{Q}$ Coefficient

For the *X*-axis, the *K*${}_{Q}$ value was 50.8618. For the *Y*-axis, the value of *K*${}_{Q}$ was 17.4666. In the *Z*-axis, *K*${}_{Q}$ was 55.7484. Using the CFD-obtained values for *K*${}_{Q}$, we determined that the error for the *X*-axis was 17.65%; for the *Y*-axis case, the error value was 39.34%; and the error value in the *Z*-axis was 4.84%. In Table 9, a comparison between the results for *K*${}_{Q}$ is shown. Additionally, in Table 10, the general results for this test are shown.

### 4.4. Free Decay Pendulum Experiment Results Using the Morison Equation

#### 4.4.1. Added Mass

For the *X*-axis, the added mass value was 8.3472 kg, with a *Cm*${}_{x}$ of 0.9073. In the *Y*-axis, the value of added mass was 2.2135 kg, with a *Cm*${}_{y}$ of 0.2406. For the *Z*-axis, the added mass was 7.6194 kg, with a *Cm*${}_{z}$ of 0.8281.

#### 4.4.2. $C_{D}$ Coefficient

For the *X*-axis, the $C_{D}$ coefficient was 2.3432. In the *Y*-axis, the $C_{D}$ coefficient was 0.5838. For the *Z*-axis, the value for $C_{D}$ was 1.1145.

**Figure 19:**
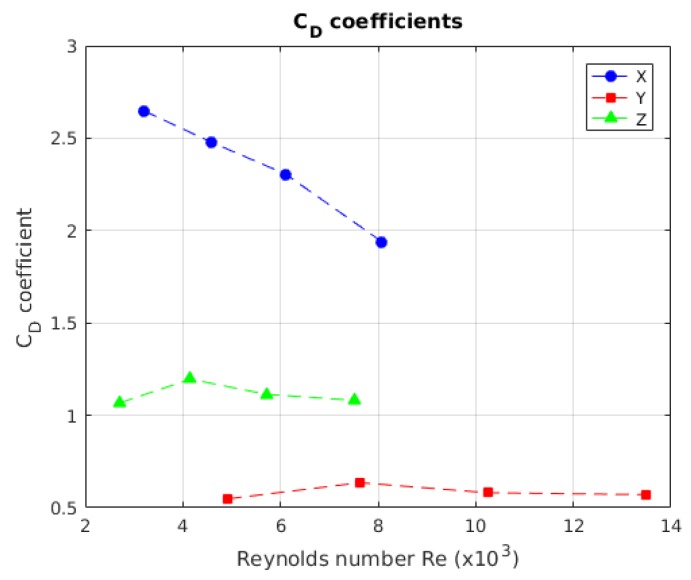
Behaviour of the drag coefficient with respect to speed. It is not possible to determine a characteristic behaviour for each axis.

In Figure 19, the behaviour of $C_{D}$ with respect to the velocities is shown, where the $C_{D}$ behaviour for the *X*-axis, had a relevant decrease. In Table 10, the results for this test are summarised.

## 5. Discussion

In this article, we obtained the values of hydrodynamic coefficients using one simulation and two experiments. The model characteristics for the identification process are mass, volume and characteristic dimensions, which are used to apply conversions to transform the scale model values to real robot values. The scale model used in each experiment has a geometric relation and, therefore, a volumetric relation to the real robot; however, the mass relationship does not correspond to the design scale as the models were made of different materials, which could provide an error source in the inertial terms.

The obtained coefficients relate forces in the same direction to the displacement; thus, the crossed values were not obtained from these methods. Fossen [28] parametrised the values of the hydrodynamic coefficients according to the geometrical symmetric conditions. The robot model analysed did not satisfy those conditions, which could be another error source, with respect to the theoretical model.

### 5.1. Added Mass Coefficient Estimation

The added mass coefficients were obtained based on the Morison equation. As the robot had an irregular shape, there exist references for determining the coefficients in different ways, in reference to its geometry. In the experiments described here, the theoretical values of added mass were not contemplated as reference values. For the drag coefficient case, the theoretical value was obtained by CFD simulation.

The added mass coefficient obtained using the free decay test was obtained with relation to the natural frequency of the oscillatory behaviour. Due to this behaviour, launches that had similar natural frequency obtained a similar added mass coefficient. In Table 6, the values that do not refuse the null hypothesis were found concentrated on the *Z*-axis, as seen in the launch combinations L${}_{1}$–L${}_{2}$, L${}_{1}$–L${}_{3}$ and L${}_{3}$–L${}_{4}$. According to the work by the authors of [32], the body frequency is affected if the oscillation is near to a wall. The relevant importance of the above-mentioned tests was due to a gap relation less than the other tests (an indicator decrease of ~66%); that is, the oscillatory moment was too near of the container wall. This case is of importance and is relevant to the robot model, as a common situation is one in which a robot is working in a recurrent way, which means that its displacement in the *Z*-axis is near a wall, such as the seabed. This is in accordance with the literature, that the gap relation is a factor that generates modification in the results. There is another indicator when the movement is oscillatory, the KC indicator (unitless), which considers the oscillatory amplitude in one dimension of the analysed model.

The added mass coefficients obtained through the free decay pendulum test were different with respect to the coefficients obtained from the free decay test. The way that the coefficients in the free decay test were obtained used a method that did not require least squares to solve it; the values were, instead, graphically extracted. For the *X*-axis, there was 10.42% relative error; for the *Y*-axis, there was 23.59% relative error (which is equivalent to 0.4222 absolute error); and, finally, for the *Z*-axis, the relative error was 28.36%. It is important to determine that, in the relative error of the *Z*-axis, the increase was due to the representative value of the mean of all values. In the work by the authors of Figure 17, it is evident that added mass values for the X- and *Z*-axis in low Reynolds conditions are equal, due to the gap effect mentioned before. If values of added mass are taken with a with gap indicator less than 1, the relative error in the *Z*-axis will be 1.49%. If the identification process is made with the method detailed in this article, the added mass value for the *X*-axis had a relative error, with respect to the free decay test experiment, of 11.02%; for the *Y*-axis, the relative error was 23.69%; and, for the *Z*-axis, the error was 28.38%. The obtained errors using the free decay pendulum test may have two different origins: the first is that the nonlinear behaviour due to the response had variations when the initial angle was bigger. The second possible error source could be that the size of the model generated other hydrodynamic effects that were not considered.

### 5.2. Damping Coefficient Estimation

The damping coefficient was obtained in the free decay test in an indirect way; this means that it was obtained from composed values as a function of the robot’s geometrical characteristics and its displacement. This means that, if the damping was similar in every launch, then the coefficient value will be similar too, as evidenced by the authors of Table 7. The theory also establishes that the values for $C_{D}$ tend to decrease when the Reynolds number increases; nevertheless, there was no way to validate that information more than assuming that just one drag force representation existed; if we assumed a quadratic coefficient, we would obtain the values in the two different methods.

According to the approximate modelling, there exists a linear coefficient in the function of the potential damping and possible superficial friction, and the values for this component contribute a significant value in lower-order Reynolds numbers. Their contribution to a high order of the linear component in CFD simulation was 1.19% for the *X*-axis, 0.98% for the *Y*-axis and 0.11% for the *Z*-axis. As the relative error was ~1%, it can be considered as an approximate error of the regression; however, there is no research in the literature where the elimination of this component has been proposed.

For the linear coefficient values obtained in the free decay pendulum test, they had high relative error with respect to the simulation, which suggests that there is a non-expected behaviour in the values obtained. The highest absolute error was 0.25; this may be because, in the pendular movement, the model experiments demonstrate hydrodynamic effects that do not appearing the simulation. Therefore, it is proposed to consider this model values over the theoretic values because they derive from real conditions.

While the quadratic coefficients showed behaviour close to the simulation, this was based on relative error. There have been several studies where the obtained coefficients for a complex shape were proposed, which allowed for determination of the obtained values as coefficients for model identification. However, in the conditions where the linear coefficient is considered for elimination, there is no option to rewrite the Morison equation; thus, this coefficient can represent more hydrodynamical effects with a quadratic relation between velocity and force, generating two different representations of the model for the hydrodynamic effects.

By obtaining the drag coefficient ($C_{D}$) from the free decay test, we can obtain the error for the obtained drag coefficients from the free decay pendulum test: for the *X*-axis, the error was 67.17%; for the *Y*-axis, the error was 15.03%; and, for the *Z*-axis, the error was 12.38%. The *X*-axis had a high error due the fact that the proportion between the front face and model length is bigger. For other cases, this proportion is lower; this makes evident that the coefficient also depends on this relation, as the X-face is the unique planar face of the model.

## 6. Conclusions

In this paper, the results of three different methods for obtaining the hydrodynamic coefficients of added mass, linear *K*${}_{L}$ and quadratic *K*${}_{Q}$ coefficients and drag coefficient $C_{D}$ are presented. From the free decay test, the values for added mass and the drag coefficient were extracted. From the free decay pendulum test, the linear and nonlinear term coefficients, drag coefficients and added mass were obtained. The values for the linear and nonlinear term coefficients and drag coefficients were also obtained using CFD simulation.

For the linear and nonlinear term coefficients obtained through the Chin Method, CFD simulation and free decay test, similar results were seen. For the exposed method in this article, using the same structure as the Morison equation, the drag coefficient values ($C_{D}$) were similar to the free decay test results, free decay pendulum test results and CFD simulation.

Values for the added mass of the robot model, using the Chin method in the free decay test, were obtained. The values were found to be similar in the X- and Y-axes, but the result for the *Z*-axis changed obviously, which could possibly be due to the gap ratio conditions in the experiment.

The values selected to be part of the model are shown in Table 10. They were selected because they are the most meaningful, due to the fact that the coefficients form two hydrodynamic coefficient models for the robot: if you want to use the Chin model, you use *K*${}_{L}$ and *K*${}_{Q}$; but, if the Morison implementation is desired, $C_{D}$ is used. The added mass coefficient is the same for both cases.

In extension of the Chin method conclusions, an identification process through free decay tests without large and costly deployments in instrumentation and for robots with complex shape has been detailed, demonstrating that using a model closer to the Morison equation does not necessarily imply more accurate values. On the other hand, there may be conditions where more effects, which were rejected in this study, should be considered.

## Figures and Tables

**Table 1:** Physical characteristics of the real robot

Dimension	Value
Weight (kg)	9.36
Volume (mm${}^{3}$)	528,797
L1 (m)	0.298
L2 (m)	0.269
L3 (m)	0.169
L4 (m)	0.348
L5 (m)	0.395
L6 (m)	0.045

The symbols for the length dimensions will be used for the description of the experimental models.

**Table 2:** Parameters of the model for the free decay test.

Parameter	Value
Weight (kg)	0.97
Volume (mm${}^{3}$)	344,868
Material	Acrylonitrile butadiene styrene (ABS) and lead
Scale	30%

The model is printed in ABS, but is filled with lead balls.

**Table 3:** Parameters of the model for the free decay pendulum test.

Parameter	Value
Weight (kg)	0.146
Volume (mm${}^{3}$)	107,597
Material	PVC
Scale	22%

**Table 4:** Summary of parameters for the Computational Fluid Dynamic (CFD) simulation configuration.

Mesh Configuration		Solver Setting	
Resolution factor	1	Solution mode	Transient
Edge growth rate	1.1	Advection scheme	Monotone streamline upwind
Layer factor	0.45	Turbulence model	k-epsilon
Layer gradation	1.05	Time step size	0.1 s
Number of nodes	46,879	Iterations	500
Number of elements	204,258	Time Stop	50 s

**Table 5:** Values Obtained through the CFD Simulation.

Axis	X	Y	Z
*K*${}_{L}$ Coefficient	1.0503	0.5706	0.1306
*K*${}_{Q}$ Coefficient	43.2322	28.7941	58.5848
R${}^{2}$	0.9960	0.9963	0.9967
*K*${}_{D}$ Coefficient	42.0253	28.1384	58.4346
$C_{D}$ Coefficient	1.7021	0.6226	1.2929
R${}^{2}$	0.9961	0.9964	0.9967

**Table 6:** Stochastic values for added mass in the free decay test.

		X	Y	Z
L${}_{i}$	L${}_{j}$	*p*-value	*p*-value	*p*-value
		*z*-value	*z*-value	*z*-value
1	2	0.1983	0.1202	0.0393 ${}^{1}$
		−1.286	−1.553	2.0603
1	3	1.0000	0.4382	0.0047 ${}^{1}$
		0	−0.775	2.8235
1	4	0.6076	0.1210	1.0000
		0.5133	−1.550	0
2	3	0.0020 ${}^{1}$	0.2470	1.0000
		3.0836	−1.157	0
2	4	0.6995	0.0717	0.5986
		−0.385	−1.8	−0.5263
3	4	0.2485	0.04 ${}^{1}$	0.0127 ${}^{1}$
		−1.153	−2.053	0

${}^{1}$ emphasises where the null hypothesis is true.

**Table 7:** Values obtained in the free decay test.

Axis	X	Y	Z
Damping Coefficient, ς	0.1971	0.109	0.1418
Drag Coefficient, $C_{D}$	1.4015	0.5075	0.9917
Natural Frequency, $\omega_{n}$ ($\frac{rad}{s}$)	7.1142	7.8268	6.8188
Added Mass Coefficient (*Cm*)	0.8172	0.1945	1.1564

**Table 8:** Stochastic values for *K*${}_{L}$ in the free decay pendulum test.

		X	Y	Z
L${}_{i}$	L${}_{j}$	*p*-value	*p*-value	*p*-value
		*z*-value	*z*-value	*z*-value
1	2	0.9362	0.8102	0.2980
		−0.080	−0.240	−1.040
1	3	0.3785	0.8102	0.2980
		−0.880	−0.240	−1.040
1	4	0.0656	0.8102	0.2280
		−1.841	−0.240	−1.201
2	3	0.3785	0.8102	0.8102
		−0.880	−0.240	−0.240
2	4	0.1735	0.8102	0.5752
		−1.361	−0.240	−0.560
3	4	0.9362	0.8102	1.0000
		−0.080	−0.240	0.0

**Table 9:** Stochastic values for *K*${}_{Q}$ in the free decay pendulum test.

		X	Y	Z
L${}_{i}$	L${}_{j}$	*p*-value	*p*-value	*p*-value
		*z*-value	*z*-value	*z*-value
1	2	0.1735	0.4712	0.2298
		−1.361	0.7205	−1.201
1	3	0.3785	0.0453	0.5752
		0.8807	2.0016	0.5604
1	4	0.6889	0.0082	0.0131 ^1^
		0.4003	2.6421	2.482
2	3	0.3785	0.0927	0.0927
		0.8807	1.6813	1.6813
2	4	0.2980	0.0131	0.0051 ${}^{1}$
		1.0408	2.482	2.8022
3	4	0.8102	0.4712	0.1735
		0.2401	0.7205	1.3611

${}^{1}$ emphasises where the null hypothesis is true.

**Table 10:** Values Obtained through the free decay pendulum test.

Axis	X	Y	Z
*K*${}_{L}$ Coefficient	1.0745	0.6508	0.3834
*K*${}_{Q}$ Coefficient	50.8618	17.4666	55.7484
$C_{D}$ Coefficient	2.3432	0.5838	1.1145
Added Mass Coefficient (*Cm*)	0.9024	0.2404	0.8283
